# Stem cell-based embryo models: The 2021 ISSCR stem cell guidelines revisited

**DOI:** 10.1016/j.stemcr.2025.102514

**Published:** 2025-06-10

**Authors:** Amander T. Clark, Heidi Cook-Andersen, Sarah Franklin, Rosario Isasi, Debra J.H. Mathews, Vincent Pasque, Peter J. Rugg-Gunn, Patrick P.L. Tam, Hongmei Wang, Jan J. Zylicz, Janet Rossant

**Affiliations:** 1 Department of Molecular Cell and Developmental Biology, Center for Reproductive Science, Health and Education, University of California, Los Angeles, Los Angeles, CA 90095, USA; 2 Department of Obstetrics, Gynecology, and Reproductive Sciences, School of Medicine, University of California, San Diego, La Jolla, CA 92093, USA; 3 Department of Molecular Biology, University of California, San Diego, La Jolla, CA 92093, USA; 4 University of Cambridge, Cambridge, UK; 5 John P. Hussman Institute for Human Genomics, Interdisciplinary Stem Cell Institute, University of Miami Leonard M. Miller School of Medicine, Miami, Florida, USA; 6 Berman Institute of Bioethics and Department of Genetic Medicine, Johns Hopkins University, Baltimore, MD 21205, USA; 7 KU Leuven-University of Leuven, Department of Development and Regeneration, Leuven Institute for Single-Cell Omics (LISCO), Leuven, Belgium; 8 Epigenetics Programme, Babraham Institute, CB22 3AT Cambridge, UK; 9 Cambridge Stem Cell Institute, Jeffrey Cheah Biomedical Centre, University of Cambridge, Cambridge, CB2 0AW, UK; 10 Loke Centre for Trophoblast Research, University of Cambridge, Cambridge, CB2 3EG, UK; 11 Children’s Medical Research Institute and School of Medical Sciences, Faculty of Medicine and Health, University of Sydney, Sydney, NSW, Australia; 12 Key State Laboratory of Organ Regeneration and Reconstruction, Institute of Zoology, Chinese Academy of Sciences, Beijing 100101, China; 13 Novo Nordisk Foundation Center for Stem Cell Medicine – reNEW, Department of Biomedical Sciences, Faculty of Health and Medical Sciences, University of Copenhagen, Copenhagen, Denmark; 14 The Gairdner Foundation and the Hospital for Sick Children, University of Toronto, Toronto, Canada

**Keywords:** stem cell-based embryo models, ISSCR guidelines, embryo

## Abstract

Human stem cell-based embryo models (SCBEMs) are a research technology with the potential to facilitate our understanding of human embryogenesis, improve assisted reproductive technology outcomes, elucidate the causes of early pregnancy failure, and provide a clearer understanding of the developmental origins of disease. Given that human SCBEMs are designed to model specific phenotypic features and developmental processes of human embryos, they raise distinct concerns from other stem cell models, such as organoids. The International Society for Stem Cell Research (ISSCR) Guidelines for Stem Cell Research and Clinical Translation, published in 2021, made recommendations for research oversight of SCBEMs and established different categories of review based on involvement of embryonic and extraembryonic lineages. However, recent progress has enabled unexpected ways to create increasingly complex models, as well as more efficient means of doing so without including all major extraembryonic lineages. A working group was tasked by the ISSCR executive to undertake a thorough reexamination of the guidelines in the light of these advances. The three main recommendations of the working group are that all research involving organized 3-dimensional human SCBEMs (1) should be subject to appropriate review, (2) must have a clear scientific rationale, and (3) must be subject to limited timelines. The proposed modifications to the ISSCR guidelines are intended to bring more clarity to the field, help guide the deliberations of researchers, oversight committees and other relevant stakeholders, and ensure continued public confidence.

## Teaser

Rossant, Clark, and colleagues were invited by the ISSCR executive to review the science of SCBEMs and to provide recommended updates to the 2021 ISSCR guidelines. This paper describes the committee’s findings and proposes new recommendations for oversight of SCBEMs. These recommendations form the basis for version 1.1 (2025) ISSCR Guidelines for Stem Cell Research and Clinical Translation.

## Introduction

Our understanding of the biology of the early human embryo is still limited. Research on embryos created by *in vitro* fertilization (IVF) that are no longer required for reproductive purposes and/or donated for research under strict regulation has provided critical knowledge regarding the cellular and molecular events of zygote to blastocyst development. Translating this knowledge to practice has contributed to enhancing the success of IVF treatments. However, the events of implantation into the uterus; development of the extraembryonic yolk sac, amnion, extraembryonic mesoderm, trophoblast, and germ cells; initiation of gastrulation with the formation of the three germ layers (ectoderm, mesoderm, and endoderm); and early patterning of the body axis, all of which are key to future successful pregnancy, and future fertility remain poorly understood. Understanding of the events of early human development beyond the blastocyst stage has been limited to *in vitro* cultures of donated blastocysts up to 14 days post-fertilization (Deglincerti et al., 2016; Molè et al., 2021; Shahbazi et al., 2016; Wu et al., 2023; Xiang et al., 2020) and analysis of rare embryonic material recovered from pregnancy terminations (Tyser et al., 2021; Xiao et al., 2024). Use of human embryos for research remains a subject of debate and is prohibited in several of jurisdictions.

There has recently been growing interest in the possibility of modeling key aspects of early human embryo development, including peri-implantation and postimplantation stages, by using pluripotent stem cell lines and their derivatives to generate human stem cell-based embryo models (SCBEMs) (Arias et al., 2022; Rossant and Tam, 2021; Shahbazi and Pasque, 2024; Wu and Fu, 2024). The ability to generate 3D *in vitro* organized models of these stages of development overcomes the limitations of access to human embryos and eases some of the ethical and regulatory concerns with the use of donated human embryos for research. The future scale and scope of studies possible with SCBEMs far exceed what can be achieved with human embryonic material itself. However, well-regulated research with human embryos will remain important to not only validate such human SCBEMs and their research outcomes but also fill critical knowledge gaps that SCBEMs currently cannot address. The combined insights gleaned from human embryos and human SCBEMs could together deliver improved outcomes of assisted reproductive technologies, better understandings of the causes of early pregnancy failure, and transformational insights into the developmental origins of many human diseases. While human SCBEMs have important applications, they also raise new ethical and regulatory concerns (Rivron et al., 2023; Nuffield Council on Bioethics, 2024).

In the process of revising the International Society for Stem Cell Reasearch (ISSCR) Stem Cell Guidelines for release in 2021, new recommendations on the oversight of SCBEMs were developed through updating the categories of review (Clark et al., 2021; Lovell-Badge et al., 2021). The 2021 ISSCR Guidelines have three major categories of review and two subcategories. A brief summary of these categories is provided here. Category 1A involves research determined to be exempt from a specialized scientific and ethics oversight process. Category 1B involves research that is reportable to the entity or body responsible for the specialized scientific and ethics oversight process. Category 2 involves forms of research that are permissible only after review and approval through a specialized scientific and ethics review process. Category 3A involves research activities currently not permitted. Category 3B involves prohibited research activities. These recommendations helped to establish an international scientific framework for the use of human SCBEMs to guide scientists, oversight committees, regulators, journals, funding agencies, and the public as the embryo modeling field grows and develops. The framework recognized that all such recommendations are subject to local legal and regulatory jurisdiction in different countries. At the time of the release of the revised guidelines in 2021, there were only a few publications on SCBEMs, which were mostly in mice, and the Guidelines Task Force thus had to anticipate future development of SCBEMs in the human context. As outlined in the following, the pace of the science exceeded their expectations, prompting the revisit and proposed revision of these guidelines outlined in the following.

## Research advances led to uncertainties in guideline interpretation

The 2021 guidelines defined an SCBEM in the glossary as “the assembly, differentiation, aggregation, or re-association of cell populations in a manner that models or recapitulates key stages of embryonic development.” The 2021 guidelines also introduced a distinction between integrated and non-integrated embryo models, on the basis that that an integrated model that included epiblast and all the extraembryonic lineages might be able to generate highly organized structures resembling the intact human embryo growing in the uterus. In particular, it was noted that inclusion of trophoblast cells in such models might lead to the future potential for implantation and placental formation. Integrated models were thus proposed to be subject to a more extensive scientific and ethical review process to justify the scientific rationale and define the limits of the developmental timeline for experimental studies. Non-integrated models were those that consisted of epiblast derivatives alone, such as gastruloids, even if they developed other extraembryonic cell types, such as amnion (Table 1).

**Table 1:** Examples of SCBEMs with current and updated recommended categories of review

Model name	Basic information	Current category	Updated category
Trophoblast or Yolk sac organoids	3D differentiation of extraembryonic lineages without inclusion of additional pluripotent cells (Castel et al., 2020; Karvas et al., 2022; Tamaoki et al., 2023; Turco et al., 2018)	1A	1A
Micropatterned colonies	Bioprinted 2D models of spatially organized postimplantation cells in micropatterned colonies (Warmflash et al., 2014).	1B	1A
PASE, embryonic-like sac	3D models of the postimplantation amniotic sac without hypoblast or trophoblast (Shao et al., 2017; Zheng et al., 2019).	1B	2
Gastruloids Somitoids Axioloids Neuruloids A-P organoids	3D models of postimplantation embryos, which mimic some aspects of embryo formation while not including derivatives of hypoblast or trophoblast (Anand et al., 2023; Haremaki et al., 2019; Miao et al., 2023; Moris et al., 2020; Sanaki-Matsumiya et al., 2022; Yamanaka et al., 2023)	1B	2
Peri-gastruloid heX-embryoid hEEs Gastruloids	3D models of peri- and early postimplantation embryos, which include one of either hypoblast or trophoblast (Hislop et al., 2024; Liu et al., 2023; Pedroza et al., 2023; Yuan et al., 2023).	1B	2
Blastoid EPS-blastoid iBlastoid hTBLC-blastoids	3D models of the blastocyst, which include hypoblast and trophoblast (Fan et al., 2021; Guo et al., 2024; Kagawa et al., 2022; Karvas et al., 2023; Li et al., 2024; Liu et al., 2020; Sozen et al., 2021; Yanagida et al., 2021; Yu et al., 2021, 2023).	2	2
E-assembloid SEM Bilaminoid Embryoid	3D models of peri-implantation and early postimplantation embryos, which include trophoblast, hypoblast, and other extraembryonic cell types (Ai et al., 2023; Okubo et al., 2024; Oldak et al., 2023; Simunovic et al., 2022; Weatherbee et al., 2023).	2	2

Names refer to the nomenclature given by the authors. PASE, postimplantation amniotic sac embryoid; A-P organoid, anterior-posterior organoid; SEM, structured stem cell-based embryo model; hEEs, human extra-embryoids; heX-embryoid, human extraembryonic embryoid; EPS, extended pluripotent stem; hTBLCs, human totipotent blastomere-like cells; E-assembloid, embryo-like assembloid.

At the time, the only human models that met the definition of an integrated model were blastoids. They were designed to mimic the blastocyst with its three founding lineages, epiblast, hypoblast, and trophoblast (Table 1). Blastoids of mice, cows, pigs, and non-human primates have so far shown limited ability to progress to organized postimplantation development *in vivo* (Kagawa et al., 2022; Li et al., 2019, 2023; Pinzón-Arteaga et al., 2023; Rivron et al., 2018; Yu et al., 2021). When cultured *in vitro* for up to 21 days after generation, some human blastoids appear to have demonstrated the capacity for organized embryo-like development (Karvas et al., 2023). However, this was still limited when compared with some of the more successful *in vitro* culture systems for non-human primate embryos (Ma et al., 2019; Zhai et al., 2023).

Since 2021, multiple studies have reported the generation of complex human SCBEMs that skip the blastocyst stage and directly mimic peri-implantation and early postimplantation development (Table 1). These models are generated from pluripotent stem cells and may or may not incorporate trophoblast and hypoblast derivatives. The capacity of such models to show organized amnion, extraembryonic mesoderm, yolk sac, and primitive streak formation, however, suggests a higher-order organization that could, with future improvements, eventually progress toward later embryo structures including heart, brain, and other organ rudiments. Similar models made with mouse cells and cultured in complex media in roller culture have demonstrated the capacity to develop to neurulation, anterior-posterior patterning, and formation of heart and somite structures (Amadei et al., 2022; Lau et al., 2022; Tarazi et al., 2022). Notably, therefore, the incorporation of trophoblast derivatives does not seem to be a prerequisite for organized development of embryo-like structures in all SCBEMs (Table 1). Consequently, if such models were to be derived without inclusion of trophoblast or hypoblast tissues, they would have to be considered as non-integrated models under the current ISSCR guidelines. Even designated non-integrated models such as gastruloids can respond to exogenous signaling cues, normally provided by extraembryonic cell types; as a result, they can develop complex embryonic structures. None of these models can in any way be considered to be biologically equivalent to the embryo itself. However, given the advances in the field, it is prudent to monitor their future development carefully, regulate accordingly, and revise the categorical distinctions developed and released in 2021.

Given the complexity and heterogeneity of more recent (post 2021) human SCBEM technologies, interpretation of the first set of ISSCR guidelines has become increasingly difficult for scientists, oversight bodies, funding agencies, regulatory authority, and journals. Furthermore, the current situation in different jurisdictions regarding the oversight of these models is complex and in flux. Following extensive discussion with a wide range of stakeholders, and informed by the recent release of the UK SCBEM Code of Practice (Cambridge Reproduction and Progress Educational Trust 2024), the current working group has concluded that the distinction between “non-integrated” versus “integrated” embryo models is no longer a distinction that will aid in effective guidance or regulation. The working group has revised the definition of a human SCBEM to be “the assembly, differentiation, aggregation, or re-association of cell populations in a manner that models or recapitulates key stages of embryonic development in 3D. These entities are designed to model specific phenotypic features and developmental processes of human embryos.”

Given this conclusion, the following recommendation is advanced:

## The working group thus proposes that all three-dimensional, spatially organized human SCBEMs should be subject to a formal scientific and ethical oversight process that is proportionate to the complexity of the model

Despite this additional level of oversight, human SCBEMs are not to be considered biologically equivalent to human embryos. Rather, this change ensures that, if the development of SCBEMs approaches more closely that of human embryos, they will be subject to an ongoing review process closer to the type of review a jurisdiction would use for research with human embryos.

This decision has several implications that will require modification of the current ISSCR guidelines. The following modifications and extensions are therefore proposed in order to clarify the 2021 ISSCR guidelines regarding SCBEM research. The outcome of our working group committee’s deliberations is designed to help guide oversight committees and other relevant stakeholders going forward by giving more clarity to the categories of review. The ISSCR is currently reviewing these recommendations, and, upon approval, these recommendations are anticipated to be included in version 1.1 (2025) ISSCR Guidelines for Stem Cell Research and Clinical Translation. In the meantime, our hope is that oversight committees and other relevant stakeholders will take our suggestions under consideration when reviewing human SCBEM research. These categories of review only apply to human SCBEMs and not SCBEMs derived from non-human cells.

## Category 1 research

In the 2021 guidelines, this category was divided into two subcategories, 1A and 1B, with non-integrated human SCBEMs classified under 1B.

Category 1A: Research that is permissible after review under existing mandates and/or committees and determined to be exempt from the specialized oversight process.

Category 1B: Research that is reportable to the oversight process but not normally subject to further review, at the discretion of the appropriate committee and/or local policy.

Whether a particular human SCBEM experiment fell under category 1A, 1B, or 2 has become increasingly unclear. We would like to propose removal of SCBEM research from category 1B and a clearer disposition of stem cell models between category 1A and category 2.

*Recommended update to category 1A*: This level of oversight will continue to include work with human pluripotent stem cell lines in culture involving routine practices such as assays of *in vitro* differentiation, spontaneous differentiation into disorganized embryoid bodies, and teratoma assays. Differentiation of bioprinted 2D cultures of pluripotent stem cells, which was previously classified as 1B, we propose should be reclassified as 1A and be exempt from specialized oversight. Trophoblast organoids, yolk sac organoids, and other structures lacking pluripotent tissue derivatives should also fall under category 1A. *In vitro* development of somatic cell or pluripotent stem cell-derived organoids, e.g., kidney organoids, liver organoids, brain organoids, etc., designed to elucidate the cellular and molecular mechanisms underpinning the development of different organ rudiments is currently included in category 1A.

*Recommended update to category 1B*: Currently this category includes *in vitro* culture of chimeric embryos generated with human pluripotent stem cells and *in vitro* gametogenesis from stem cells that does not involve fertilization. These remain under this category of “reportable but not normally subject to further review.” However, we propose that “research that entails the *in vitro* formation of human SCBEMs that are not intended to represent the integrated development of the entire embryo including its extraembryonic membranes” be removed from this section and moved to category 2.

## Category 2 research

Category 2: Forms of research with embryos and embryo models that are permissible only after review and approval through a specialized scientific and ethics review process.

In the current version of the guidelines, category 2 review of human SCBEMs was restricted to “integrated” SCBEMs that included extraembryonic cell types. However, given the complexity of development of current models with or without extraembryonic cell types and the uncertainties around the potential future development of various model systems, it is recommended that all human SCBEMs that include pluripotent stem cell derivatives with or without extraembryonic lineages and that show 3D spatial organization designed to resemble human embryos be subject to review. Human models involving a period of time in 2D culture before generating the SCBEM in 3D (Hislop et al., 2024) should also be evaluated under category 2 review. These changes will remove the difficulties faced by investigators and review panels in deciding on the appropriate review category for a given model system.

*Recommended update to category 2*: Review by an appropriate specialized committee should occur for all approaches that lead to spatially organized 3D human SCBEMs. Absence of trophoblast or other extraembryonic cell types is not considered a criterion for exclusion from review. The extent of review should be proportionate to the degree of complexity of the model. Review should include evaluation of the scientific rationale for the use of the particular model, the reasons why a less complex model is not appropriate, and a proposed time limit for the culture, related to the developmental milestones under study (Jonlin et al., 2025). Scientists are urged to liaise with their appropriate local or national oversight committee while designing their experimental protocols. An iterative approval process should be established, requiring regular reporting to the committee as experiments proceed. A request to extend the time limit for *in vitro* culture can be considered only if the committee is duly updated about ongoing experiments. An open-ended time limit for culture and experimentation of SCBEMs in this category is never appropriate. *In vitro* bioengineering approaches including the co-culture of human SCBEMs with uterine cells or uterine organoids or other culture devices would be allowable under category 2 so long as the time limit for culture based on developmental milestones is clearly stated and the experimental justification does not violate category 3B. Isolating single cells from SCBEMs for assessment of function by transplantation to an animal host (for example, hematopoietic stem cells) should be reviewed under category 2. Table 1 provides some examples of different kinds of SCBEMs and how they would be categorized under the proposed changes.

## Category 3 research

Category 3A: Research activities currently not permitted. Research under this category should not be pursued at this time because the approaches are currently unsafe or raise unresolved ethical issues.

Category 3B: Prohibited research activities. Research under this category should not be pursued because of broad international consensus that such experiments lack a compelling scientific rationale and are widely considered to be unethical.

There are a number of different stem cell and embryo research activities listed under category 3A and 3B. Relevant to SCBEMs, transfer of any human SCBEM to the uterus of a living human or animal host to continue development *in vivo* falls under prohibited research activities in category 3B. This applies to all SCBEMs regardless of the complexity of the model under study. This limits the concern that the public may have about potential future reproductive potential of such models. However, there are emerging technologies around in vitro artificial placental systems and extension of viability *ex utero* of premature babies to earlier and earlier stages that warrant ongoing discussion.

Current SCBEMs developed in research settings are designed solely for the limited-term study of fundamental processes involved in human development. There are commercial and other groups raising the possibility of building an embryo *in vitro* and combining different bioengineering approaches to bring such an entity to viability. Currently the practice of bringing an SCBEM to viability is considered unsafe and unethical and should not be pursued. The issues raised by such approaches deserve a more widespread discussion and public engagement.

This leads us to propose an addition to this restriction that would include the use of *ex vivo* artificial systems to promote development of SCBEMs closer to viability—so-called ectogenesis.

*Recommended update to category 3B:* Transfer of human SCBEMs to the uterus of a living human or animal host or culture in an artificial *in vitro* system designed to develop SCBEMs to viability (i.e., ectogenesis). This restriction would apply to all projects *for any purpose: reproductive, research, or commercial.*

## Conclusions and future issues

It is recommended that research proposals and publications, including preprints, involving SCBEMs include a statement that demonstrates the lead investigator’s understanding of the legislation and regulatory policies on research using human SCBEMs in the jurisdiction where the research work is undertaken and a statement on the applicable ISSCR guideline category. For work falling under category 1, information on the scientific review process and the basis for the determination of category 1 oversight should be provided. For work falling under category 2, information on the process of scientific review and oversight should be provided together with a statement of ethics compliance, a statement addressing why a less complex model is not appropriate to achieve the proposed goals, and the approved length of time in culture for experimentation on the model.

Research on human SCBEMs is not undertaken to generate embryos for the purposes of human reproduction. Instead, the goal of this research is to provide important insights into normal pregnancy, human development, and developmental origins of disease. All such experiments are performed using short-term *in vitro* model systems only, and we propose that they be regulated as such. Such models will not completely replace the need for research using human embryos. However, SCBEMs are expected to reduce the number of human embryos used for research. Given the apparent ease in generating large numbers of SCBEMs, they may be used to rapidly screen for improved *in vitro* culture media and embryo modifications applicable to clinical IVF practice and assisted reproductive technologies in general. However, final assessment of such discoveries would still need to be tested on human embryos donated to research before use for clinical applications. SCBEMs may also be used to screen for novel drug therapies and to assess potential embryotoxicity. Extended culture of SCBEMs will also be an important testing ground for safety of novel therapies, including genetic modification, before considering adoption of these approaches in the IVF clinic.

Currently there is no SCBEM that completely and reproducibly mimics the entirety of normal human development. However, given that the validity of such models depends on their proximity to embryos, we can expect a focus on technical improvements in the near future leading to models with closer alignment to normal embryonic development. There have been various discussions about the potential of human SCBEMs to reach a point whereby they should be considered as equivalent to embryos that are the product of fertilization (Rivron et al., 2023). As this field moves forward, it is possible that increasingly complex embryo models could develop into stem cell-based models of later embryo or even fetal development. Development of SCBEMs into the fetal stages would raise legitimate concerns, not only within the scientific community but also in the general public and regulatory authorities. Further ethical reflection and robust public engagement on the acceptability of future research using these complex models will be essential. Such engagement should include an explanation of the scientific and medical benefits of human embryo and SCBEM research up to that point and the scientific rationale (and underlying values) for allowing further stages of development. The engagement must be part of a process that meaningfully integrates public input generated through engagement into governance decisions, as has been used in the development of the UK SCBEM Code of Practice. Maintaining public trust and confidence in science requires thoughtful engagement and the balancing of scientific and societal values.

## Acknowledgments

The authors would like to thank the staff of the 10.13039/100019448ISSCR, in particular Tyler Lamb, for support. In addition, the authors would like to thank Dr Xiaomei Zhai from the School of Population Medicine and Public Health, Chinese Academy of Medical Sciences & Peking Union Medical College, Beijing, People’s Republic of China, for helpful discussions during the early phases of the working group deliberations.

## Declaration of interests

A.T.C. is an Officer of the ISSCR. J.R. is the Editor-in-Chief of Stem Cell Reports and an Ex Officio Board of Director of the ISSCR. V.P. has filed the following patent application: PCT/EP2023/073949. P.P.LT. is a member of the NHMRC Embryo Research Licensing Committee in Australia. J.J.Z has filed the following patent application: PCT/EP2025/057066 and is supported by a grant from the NNF (NNF21CC0073729).
